# A Common Ancestral Mutation in *CRYBB3* Identified in Multiple Consanguineous Families with Congenital Cataracts

**DOI:** 10.1371/journal.pone.0157005

**Published:** 2016-06-21

**Authors:** Xiaodong Jiao, Firoz Kabir, Bushra Irum, Arif O. Khan, Qiwei Wang, David Li, Asma A. Khan, Tayyab Husnain, Javed Akram, Sheikh Riazuddin, J. Fielding Hejtmancik, S. Amer Riazuddin

**Affiliations:** 1 Ophthalmic Genetics and Visual Function Branch, National Eye Institute, National Institutes of Health, Bethesda, MD, 20892, United States of America; 2 The Wilmer Eye Institute, Johns Hopkins University School of Medicine, Baltimore, MD, 21287, United States of America; 3 National Centre of Excellence in Molecular Biology, University of the Punjab, Lahore, 53700, Pakistan; 4 King Khaled Eye Specialist Hospital, Riyadh, 12329, Saudi Arabia; 5 Allama Iqbal Medical College, University of Health Sciences, Lahore, 54550, Pakistan; 6 National Centre for Genetic Diseases, Shaheed Zulfiqar Ali Bhutto Medical University, Islamabad, Pakistan; Sun Yat-sen University, CHINA

## Abstract

**Purpose:**

This study was performed to investigate the genetic determinants of autosomal recessive congenital cataracts in large consanguineous families.

**Methods:**

Affected individuals underwent a detailed ophthalmological examination and slit-lamp photographs of the cataractous lenses were obtained. An aliquot of blood was collected from all participating family members and genomic DNA was extracted from white blood cells. Initially, a genome-wide scan was performed with genomic DNAs of family PKCC025 followed by exclusion analysis of our familial cohort of congenital cataracts. Protein-coding exons of *CRYBB1*, *CRYBB2*, *CRYBB3*, and *CRYBA4* were sequenced bidirectionally. A haplotype was constructed with SNPs flanking the causal mutation for affected individuals in all four families, while the probability that the four familial cases have a common founder was estimated using EM and CHM-based algorithms. The expression of *Crybb3* in the developing murine lens was investigated using TaqMan assays.

**Results:**

The clinical and ophthalmological examinations suggested that all affected individuals had nuclear cataracts. Genome-wide linkage analysis localized the causal phenotype in family PKCC025 to chromosome 22q with statistically significant two-point logarithm of odds (LOD) scores. Subsequently, we localized three additional families, PKCC063, PKCC131, and PKCC168 to chromosome 22q. Bidirectional Sanger sequencing identified a missense variation: c.493G>C (p.Gly165Arg) in *CRYBB3* that segregated with the disease phenotype in all four familial cases. This variation was not found in ethnically matched control chromosomes, the NHLBI exome variant server, or the 1000 Genomes or dbSNP databases. Interestingly, all four families harbor a unique disease haplotype that strongly suggests a common founder of the causal mutation (p<1.64E-10). We observed expression of *Crybb3* in the mouse lens as early as embryonic day 15 (E15), and expression remained relatively steady throughout development.

**Conclusion:**

Here, we report a common ancestral mutation in *CRYBB3* associated with autosomal recessive congenital cataracts identified in four familial cases of Pakistani origin.

## Introduction

Congenital cataracts are the leading cause of vision loss in children globally, and it is estimated that one-third of blindness in infants is caused by cataracts [1,2]. It is further estimated that 1 to 6 cases per 10,000 live births develop non-syndromic cataracts in industrialized countries, whereas these figures are assumed to be much higher in developing countries [3,4]. The ocular lens focuses light on the retina and the development of cataracts interferes with this function, leading to permanent blindness, especially during early developmental periods.

Congenital cataracts are both clinically and genetically heterogeneous, manifesting both as an autosomal dominant and recessive trait. Autosomal recessive congenital cataracts (arCC) have been associated with loci and/or genes on chromosomes 1p, 1q, 3p, 3q, 6p, 7q, 8p, 9q, 11q, 16q, 17q, 19q, 20p, 21p, and 22q [5–21]. Among these, pathogenic mutations have been reported in EPH receptor A2 (*EPHA2)*, connexin50 (*GJA8)*, FYVE and coiled-coil domain containing 1 (*FYCO1*), glucosaminyl (N-acetyl) transferase 2 (*GCNT2)*, acylglycerol kinase (*AGK*), tudor domain containing 7 (*TDRD7*), crystallin alpha B (*CRYAB*), heat-shock transcription factor 4 (*HSF4*), galactokinase 1 (*GALK1*), lens intrinsic membrane protein 2 (*LIM2*), beaded filament structural protein 1 (*BFSP1*), crystallin alpha A (*CRYAA*), lanosterol synthase (*LSS*), crystallin beta B1 (*CRYBB1*), and crystallin beta B3 (*CRYBB3*) [6,7,10,14–16,18–26].

Crystallins are the most abundant proteins in the ocular lens and constitute up to 95% of the total soluble lens proteins [27]. Crystallins are classified as alpha, beta or gamma, depending upon their elution characteristics during gel exclusion chromatography [28]. The high concentration of tightly packed crystallin proteins is necessary for transparency [27]. We previously reported a missense mutation in *CRYBB3* in two familial cases of Pakistani origin [21]. As a follow-up, we now report four additional familial cases harboring the same missense mutation in *CRYBB3*.

## Materials and Methods

### Recruitment and Clinical Assessment

A total of >200 consanguineous Pakistani families with non-syndromic cataracts were recruited to identify new disease loci responsible for inherited ocular diseases. The Institutional Review Board (IRB) of the National Centre of Excellence in Molecular Biology (Lahore, Pakistan), the National Eye Institute (Bethesda, MD), and Johns Hopkins University (Baltimore, MD) approved this study. All participating family members provided informed written consent that has been endorsed by the respective IRBs and is consistent with the tenets of the Declaration of Helsinki.

A detailed clinical and medical history was obtained from the individual families. The ophthalmic examination was performed using a slit-lamp. All participating members voluntarily provided a blood sample of approximately 10 ml, which was stored in 50 ml Sterilin^®^ Falcon tubes containing 400 μl of 0.5 M EDTA. Blood samples were stored at -20°C for long-term storage.

### Genomic DNA Extraction

The genomic DNAs were extracted from white blood cells using a non-organic modified procedure as described previously [29]. The concentration of the extracted genomic DNA was estimated using a SmartSpec Plus Bio-Rad Spectrophotometer (Bio-Rad, Hercules, CA).

### Genome-Wide Scan and Exclusion Analysis

Applied Biosystems MD-10 linkage mapping panels (Applied Biosystems, Foster City, California) were used to complete a genome-wide scan for family PKCC025. Multiplex polymerase chain reaction (PCR) was completed as described previously [29]. PCR products were mixed with a loading cocktail containing HD-400 size standards (Applied Biosystems) and resolved in an Applied Biosystems 3100 DNA Analyzer. Genotypes were assigned using the Gene Mapper software from Applied Biosystems. Exclusion analyses were completed for PKCC063, PKCC131, and PKCC168 using closely spaced STR markers. The sequences of the primer pairs used for exclusion analysis and amplification conditions are available upon request.

### Linkage Analysis

Linkage analysis was performed with alleles of PKCC025 obtained through the genome-wide scan and alleles of PKCC063, PKCC131, and PKCC168 obtained through exclusion analysis using the FASTLINK version of MLINK from the LINKAGE Program Package [30,31]. Maximum LOD scores were calculated using ILINK from the LINKAGE Program Package. arCC was investigated as a fully penetrant disorder with an affected allelic frequency of 0.001.

### Mutation Screening

The sequences of the primers used to amplify *CRYBB1*, *CRYBB2*, *CRYBB3*, and *CRYBA4* are available upon request. PCR reactions were completed in 10 μl volumes containing 20 ng of genomic DNA. PCR amplification consisted of a denaturation step at 95°C for 5 min followed by a two-step touchdown procedure. The first step of 10 cycles consisted of denaturation at 95°C for 30 seconds, followed by a primer set-specific annealing for 30 seconds (annealing temperature decreased by 1°C per cycle) and elongation at 72°C for 45 seconds. The second step of 30 cycles consisted of denaturation at 95°C for 30 seconds followed by annealing (annealing temperature -10°C) for 30 seconds and elongation at 72°C for 45 seconds, followed by a final elongation at 72°C for 5 minutes.

The PCR primers for each exon were used for bidirectional sequencing using the BigDye Terminator Ready Reaction mix, according to the manufacturer’s instructions. The sequencing products were resolved on an ABI PRISM 3100 DNA analyzer (Applied Biosystems), and the results were analyzed using Applied Biosystems SeqScape software.

### Evolutionary Conservation

Evolutionary conservation of the amino acid glycine at position 165 (Gly165) in other CRYBB3 orthologs was investigated by aligning the protein sequence of CRYBB3 orthologs. The degree of evolutionary conservation of amino acid positions and the possible effect of amino acid substitution on the structure of the CRYBB3 protein were examined using SIFT (http://sift.jcvi.org) and PolyPhen2 (http://genetics.bwh.harvard.edu/pph2/index.shtml), respectively.

### Estimating the Likelihood of a Common Founder Effect

A total of 10 SNPs within 100 kb of *CRYBB3* were selected, and an affected individual from each family was genotyped to construct the causal haplotype. Primer pairs and the amplification conditions for genotyping are available upon request. SNP genotypes of 96 individuals of Pakistani descent were obtained from the 1000 Genomes database and were used to construct ethnically matched control haplotypes. Haplotype frequencies were estimated using EM and CHM algorithms as implemented in the Golden Helix SVS, and were then used to calculate the likelihood of the common founder effect.

### Real-Time Expression Analysis

The use of mice in this study was approved by the Johns Hopkins Animal Care and Use Committee (ACUC), and all experiments were performed in accordance with a protocol approved by the Johns Hopkins ACUC. Mouse lenses were obtained at different developmental stages, including embryonic day 15 (E15), day 18 (E18), at birth, designated as (P0), postnatal day 3 (P3), day 6 (P6), day 9 (P9), day 12 (P12), day 14 (P14), day 21 (P21), day 28 (P28), day 42 (P42), and day 56 (P56). Mice were first anesthetized with isoflurane and subsequently euthanized through cervical dislocation. The ocular tissue was extracted and the lenses were isolated from the retinas using forceps under a microscope. The lenses were divided into two pools, each representing a biological replicate for the respective developmental stage. Lenses were dissolved in TRIzol reagent (Invitrogen, Carlsbad, CA) immediately after extraction and total RNA was isolated from each pool according to the manufacturer’s instructions. The quality and quantity of the total RNA were determined on a NanoDrop Lite Spectrophotometer (Thermo Scientific, Inc.). First-strand cDNA synthesis was completed using the Superscript III kit (Invitrogen) according to the manufacturer’s instructions. Quantitative real-time PCR was performed on the STEP ONE ABI Real-Time PCR System using predesigned *Crybb1*, *Crybb2*, and *Crybb3* TaqMan expression assays (Applied Biosystems). *Gapdh* was used as an endogenous internal control. The 2^-ΔCT^ method was used to determine the relative expression, normalized to *Gapdh* expression, at each developmental stage.

## Results

**Fig 1:**
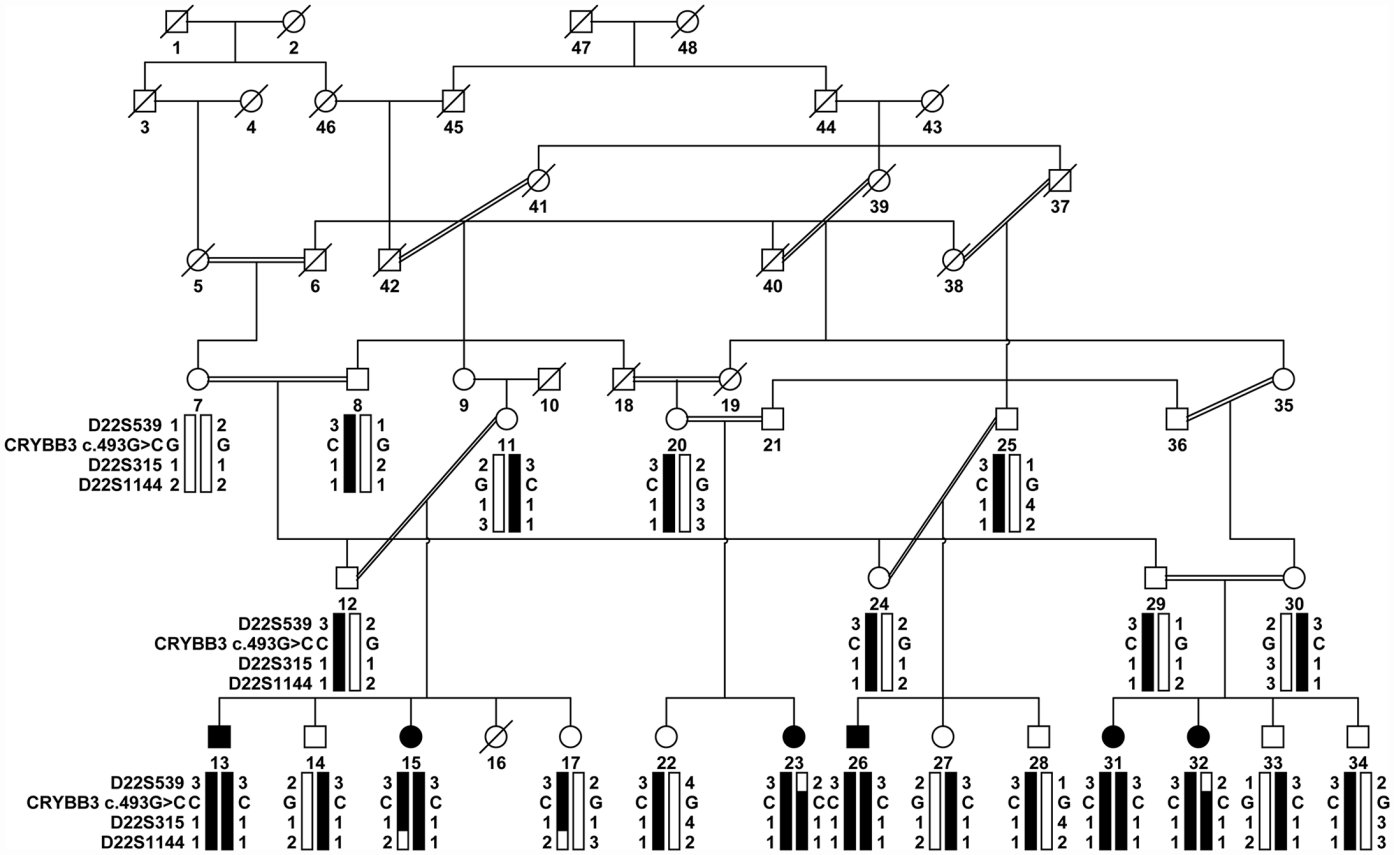
Pedigree drawing of family PKCC025 with haplotypes of chromosome 22q microsatellite markers. Alleles forming the risk haplotype are shaded black, and alleles not co-segregating with cataracts are shown in white. Squares: males; circles: females; filled symbols: affected individuals; double line between individuals: consanguineous mating; and a diagonal line through a symbol: deceased person.

**Fig 2:**
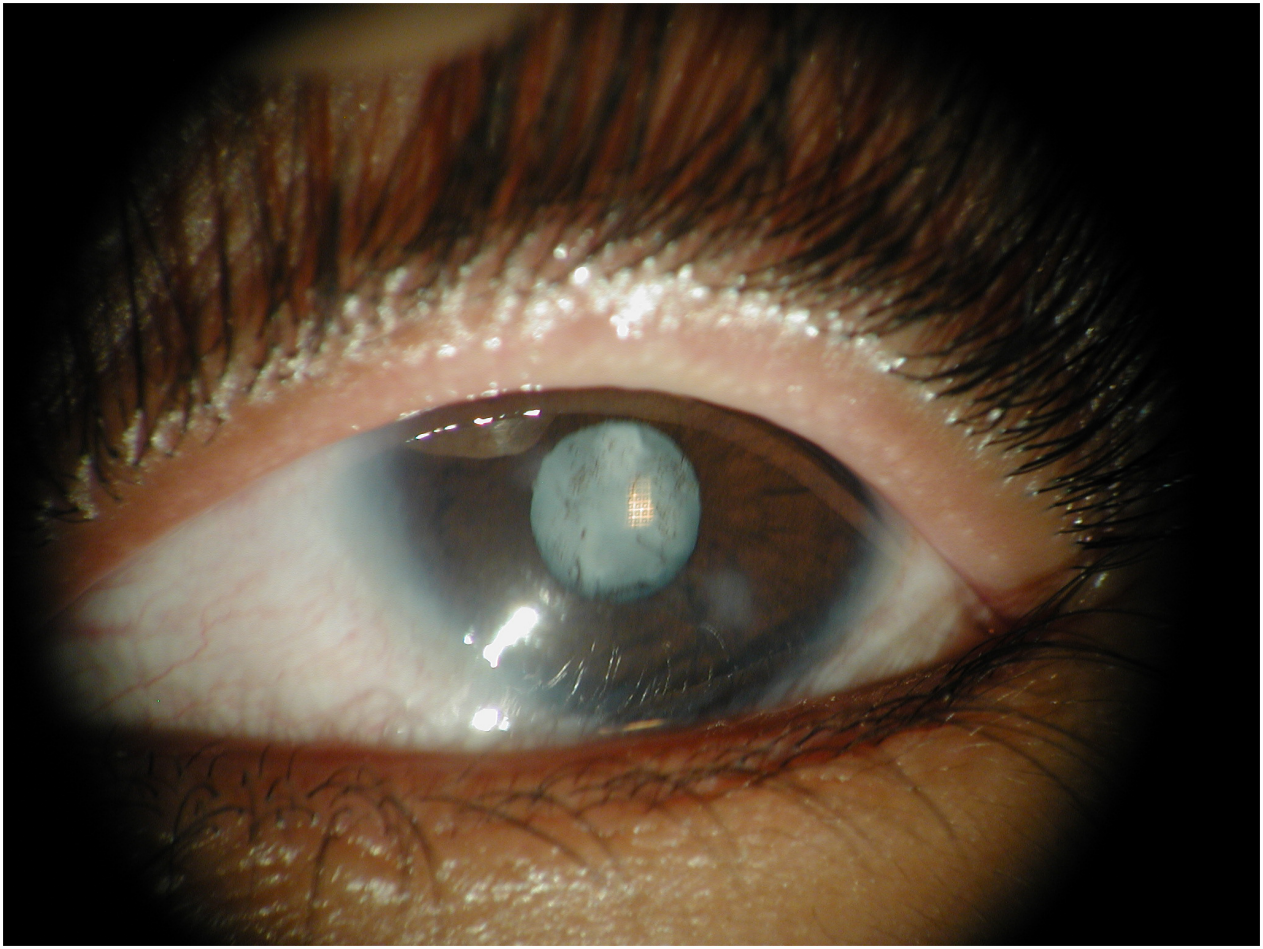
Slit lamp photograph illustrating nuclear cataracts present in affected individual 15 of family PKCC025.

A large consanguineous family, PKCC025, consisting of six affected individuals in four consanguineous marriages, was recruited from the Punjab province of Pakistan (Fig 1). From this family, we were able to enroll six affected individuals along with 16 unaffected family members. The clinical records and medical history of participating members ruled out any systemic abnormalities and/or extraocular anomalies. Clouding of the lens in all affected individuals was apparent within the first year of their life, suggesting an early, perhaps congenital, onset. A clinical examination was conducted using slit lamp microscopy, which revealed nuclear cataracts (Fig 2).

Initially, we estimated the highest theoretically possible LOD score and found that PKCC025 can attain a maximum two-point LOD of 5.15 (at ɵ = 0). Next, we completed a genome-wide scan using Applied Biosystems MD-10 linkage mapping panels that have an average spacing of 10 cM and calculated the two-point LOD scores using alleles of STR markers. We observed a possible linkage interval on chromosome 11p, containing D11S1338, yielding a two-point LOD score of 3.1 (at ɵ = 0). However, the uninformative alleles of D11S1338 and highly negative two-point LOD scores produced by D11S4046, proximally, and D11S902, distally of D11S1338, argued against linkage to chromosome 11p. We did not observe any significant two-point LOD scores (LOD >3.0) besides D11S1338 during the genome-wide scan.

We next searched for intervals of suggestive linkage (LOD > 2.0) and found three regions, first on chromosome 3p (D3S3681), second on chromosome 8p (D8S1771), and third on chromosome 10p (D10S547). Like D11S1338, markers at the proximal and distal ends of D8S1771 and D10S547, and the proximal marker of D3S3681, yielded highly negative LOD scores while a marker on the distal end of D3S33681 generated a positive signal. To further investigate the signal on chromosome 3p (D3S3681), we constructed a haplotype with alleles of STR markers adjacent to D3S3681. Visual inspection of the haplotype failed to identify a disease allele unique to the affected individuals in the family.

The lack of two-point LOD scores mimicking the theoretical potential of PKCC025 along with the exclusion of the regions of significant and/or suggestive linkage prompted us to consider the possibility that the high degree of consanguinity in PKCC025 might have reduced the critical disease interval below the 10 cM resolution of the MD-10 panel. Thus, we re-examined the genome-wide linkage data and identified a region on chromosome 22q with two adjacent panel markers, D22S539 and D22S315, yielding positive two-point LOD scores (Table 1).

**Table 1:** Two-point LOD scores yielded by chromosome 22q markers for families PKCC025, PKCC063, PKCC131, and PKCC168. Asterisk indicates markers included in the genome-wide scan.

Marker	cM	Mb	Families	0.00	0.01	0.05	0.10	0.20	0.30	0.40	*Z*_max_	ɵ_max_
D22S539*	14.44	21.9	PKCC025	-∞	2.58	2.87	2.68	2.04	1.32	0.61	2.87	0.05
			PKCC063	-1.41	1.24	1.67	1.61	1.19	0.65	0.18	1.67	0.05
			PKCC131	1.12	1.10	0.99	0.86	0.59	0.35	0.15	1.12	0.00
			PKCC168	-∞	-0.53	0.64	0.94	0.92	0.63	0.30	0.94	0.10
***CRYBB3* (c.493G>C)**		25.2	PKCC025	5.29	5.17	4.70	4.10	2.92	1.78	0.77	5.29	0.00
			PKCC063	4.84	4.76	4.42	3.98	3.05	2.06	1.02	4.84	0.00
			PKCC131	1.93	1.89	1.73	1.53	1.13	0.73	0.35	1.93	0.00
			PKCC168	3.99	3.92	3.65	3.30	2.52	1.66	0.75	3.99	0.00
D22S315*	21.47	25.6	PKCC025	2.12	2.07	1.85	1.59	1.05	0.55	0.15	2.12	0.00
			PKCC063	2.30	2.25	2.05	1.79	1.26	0.76	0.33	2.30	0.00
			PKCC131	1.44	1.41	1.27	1.09	0.74	0.42	0.17	1.44	0.00
			PKCC168	-2.32	1.20	1.67	1.67	1.33	0.86	0.39	1.67	0.05
**D22S1144**	27.48	27.2	PKCC025	-∞	2.21	2.06	1.52	0.95	0.45	1.87	2.21	0.01
			PKCC063	-∞	1.65	2.05	1.98	1.50	0.88	0.30	2.05	0.05
			PKCC131	1.59	1.56	1.42	1.25	0.90	0.55	0.24	1.59	0.00
			PKCC168	-7.87	-2.29	-0.87	-0.32	0.05	0.10	0.04	0.10	0.30

**Fig 3:**
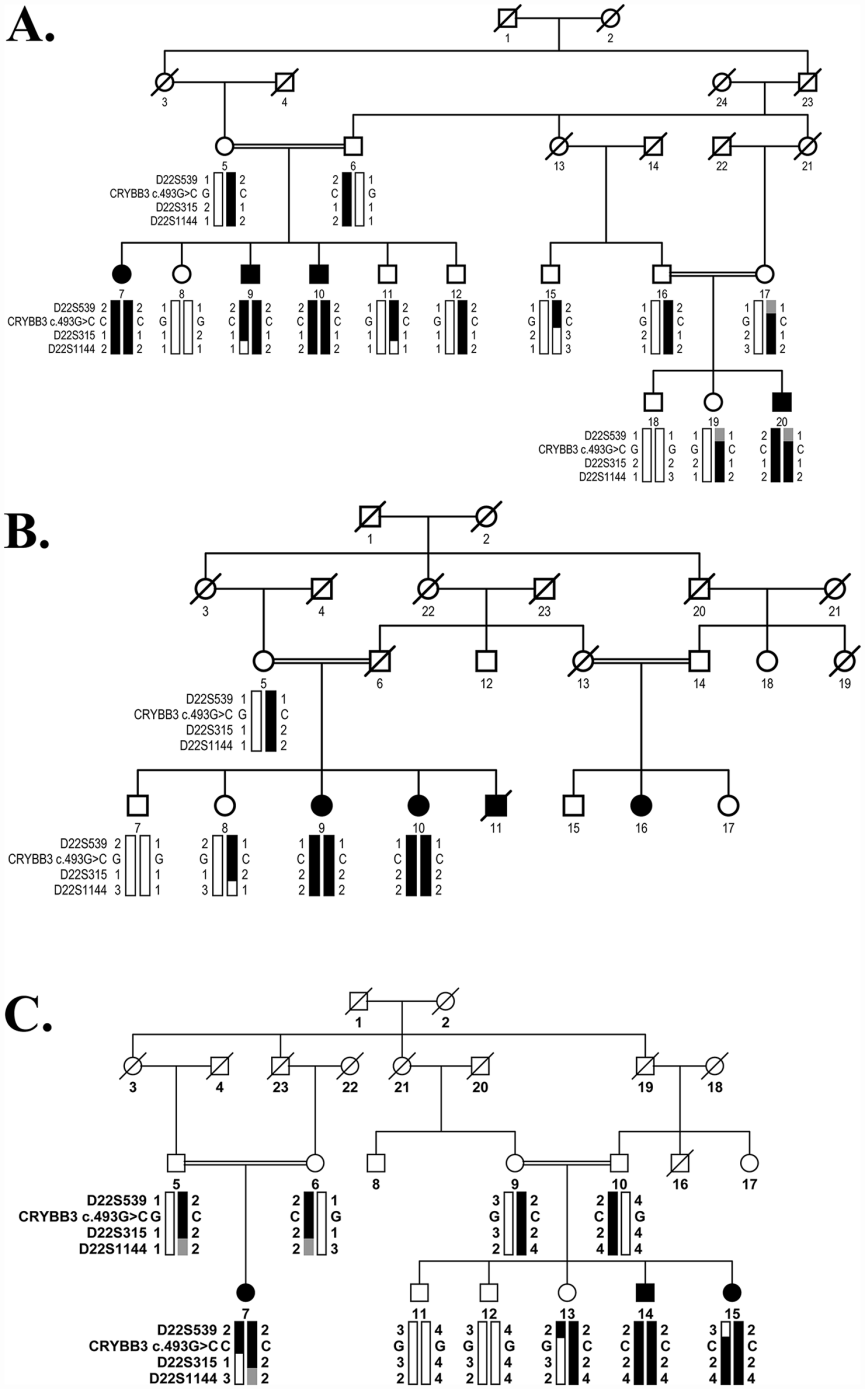
Pedigree drawing of families with haplotypes of chromosome 22q microsatellite markers. A) PKCC063, B) PKCC131, and C) PKCC168 with alleles forming the risk haplotype shaded black, alleles forming the risk haplotype but not homozygous in affected individuals shaded gray, and alleles not co-segregating with cataracts shown in white. Symbols are as described in Fig 1.

Next, we interrogated our entire cohort of >200 familial cases of congenital cataracts with closely spaced STR markers flanking the linkage interval on chromosome 22q. Visual inspection of the haplotype (constructed using alleles of the STR markers) identified three additional families (PKCC063, PKCC131, and PKCC168) linked to the chromosome 22q (Fig 3). The linkage of these three familial cases to the chromosome 22q region was further supported by positive two-point LOD scores (Table 1).

**Fig 4:**
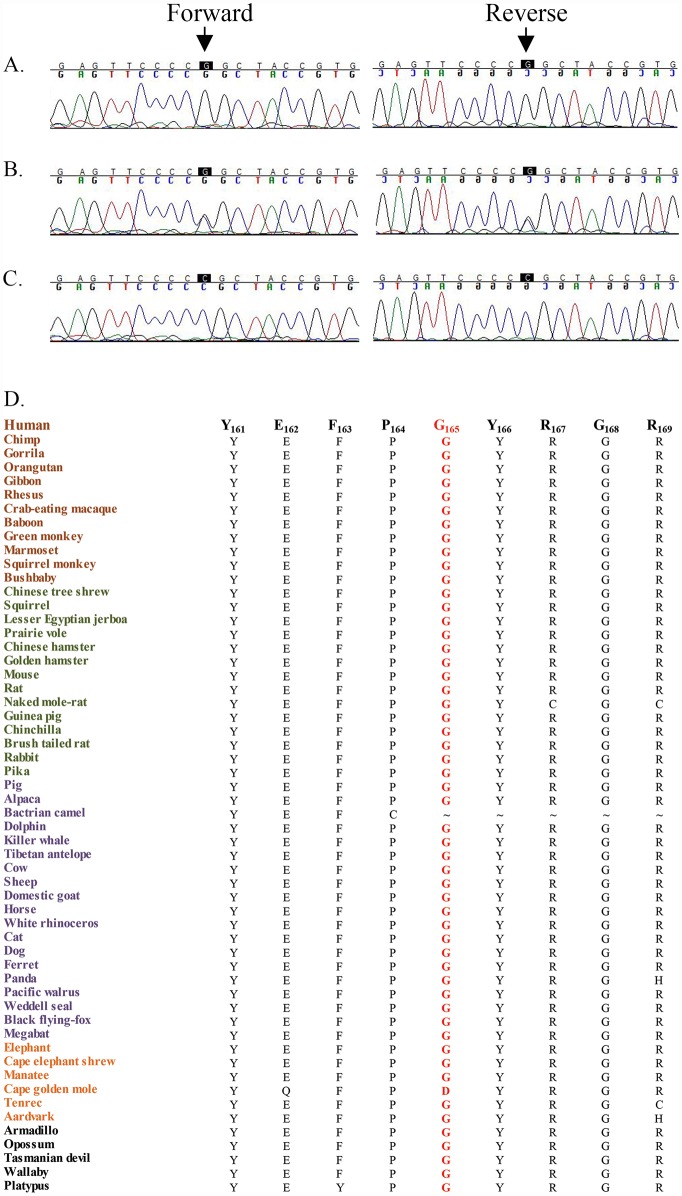
Sequence chromatograms of the c.493G>C variation identified in family PKCC025. A) Unaffected individual 7, homozygous for the wild-type allele; B) unaffected individual 8, heterozygous; and C) affected individual 15, homozygous for c.493G>C (p.Gly165Arg). D) Illustration of conservation of Gly165 in mammalian *CRYBB3* orthologs. Primates, Euarchontoglires, Laurasiatheria, Afrotheria and Mammals are colored brown, green, purple, orange and black respectively.

**Fig 5:**
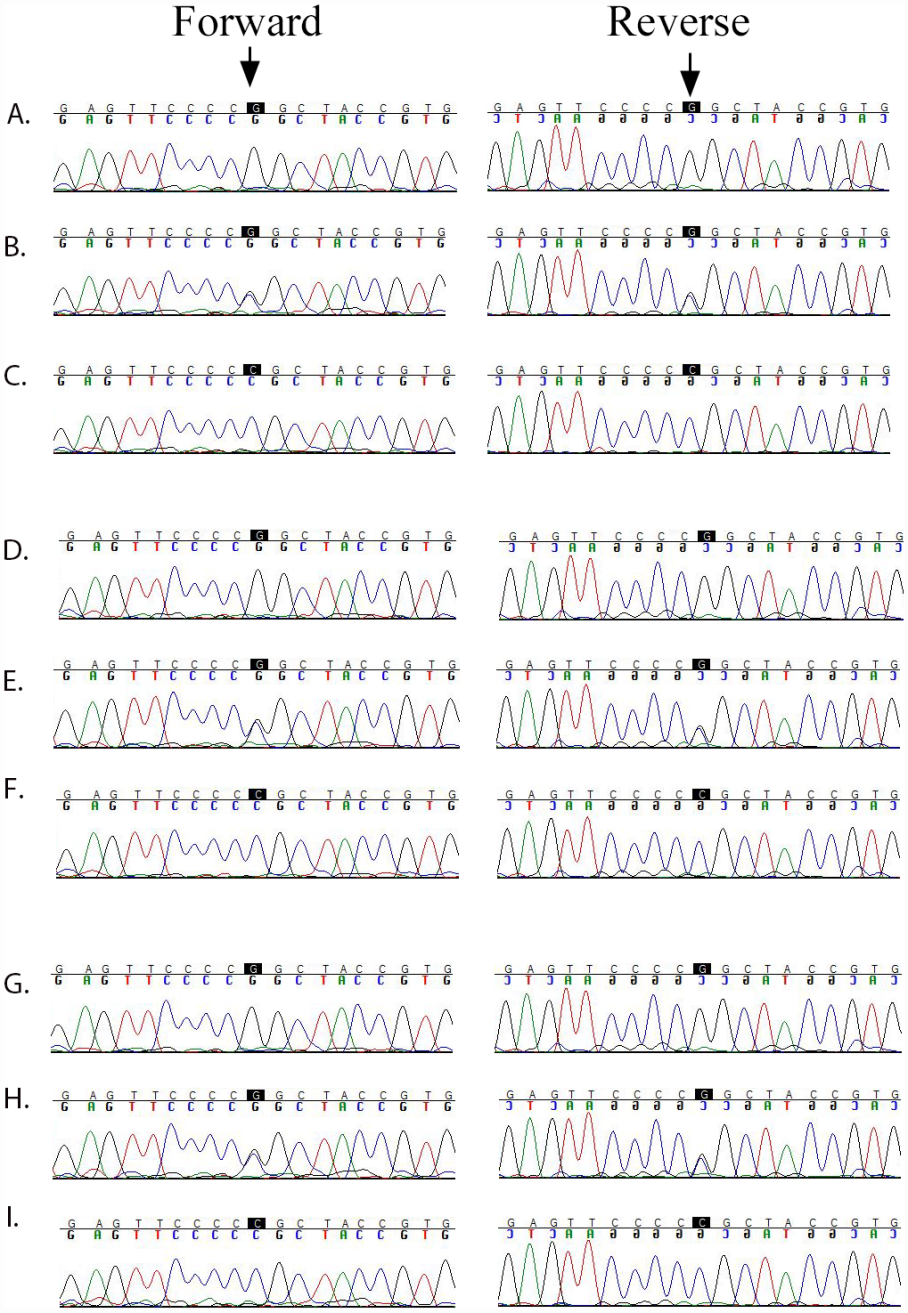
Sequence chromatograms of the c.493G>C (p.Gly165Arg) variation identified in families PKCC063, PKCC131, and PKCC168. A), D), & G) individuals 18, 7, and 12 of PKCC063, PKCC131, and PKCC168, respectively, homozygous for the wild-type allele; B), E), & H) individuals 19, 8, and 13 of PKCC063, PKCC131, and PKCC168, respectively, heterozygous, and C), F), & I) individuals 20, 9, and 14 of PKCC063, PKCC131, and PKCC168, respectively, homozygous for c.493G>C (p. Gly165Arg).

This region harbors a cluster of crystalline genes, *CRYBB1*, *CRYBB2*, *CRYBB3*, and *CRYBA4*. Bidirectional Sanger sequencing identified the missense variation c.493G>C (p.Gly165Arg) in *CRYBB3* that segregated with the disease phenotype in PKCC025 (Fig 4A–4C). It is worth noting that the Gly165 amino acid is highly conserved in CRYBB3 orthologs (Fig 4D). Importantly, the same missense variation (c.493G>C) was identified in the other three familial cases (PKCC063, PKCC131, and PKCC168) linked to the chromosome 22q (Fig 5). The c.493G>C variant was not found in 384 control chromosomes of Pakistani decent and 48 control chromosomes of Saudi Arabian decent. Additionally, this mutation was not present in the 1000 Genomes database, the NHLBI exome variant server, or the dbSNP database. Significant two-point LOD scores with the variant allele (c.493G>C) further confirmed linkage of all four familial cases (PKCC025, PKCC063, PKCC131, and PKCC168) to *CRYBB3* (Table 1). No potential variants were identified in *CRYBB1*, *CRYBB2*, and *CRYBA4* in the genomic DNAs of all four familial cases.

Interestingly, we had previously identified the same missense variation (c.493G>C) in two familial cases (PKCC004 and PKCC006) of Pakistani origin [21]. We had further shown, through molecular modeling, that changes in electrostatic potential can potentially reduce the stability of the fourth ‘Greek-Key’ motif, and hence the entire CRYBB3 protein, drastically [21]. Here, we utilized the PolyPhen2 and SIFT algorithms to assess the impact of the G165R substitution on the structure and function of CRYBB3. PolyPhen2 predicts the possible effects of an amino acid substitution on the structure and function using physical and comparative considerations. According to PolyPhen2 estimates, the p.G165R mutation is probably damaging. Likewise, SIFT prediction is based on the degree of conservation of amino acid residues in sequence alignments derived from closely related sequences. SIFT algorithms suggested that no amino acid substitution, including arginine for glycine at position 165, will be tolerated by CRYBB3.

All four families (PKCC025, PKCC063, PKCC131, and PKCC168) investigated in this study were recruited from the Punjab province of Pakistan; they reside in different cities with no known relationship between them. Thus, we wondered if c.493G is a mutational hot spot that is frequently mutated in the Pakistani population, or if these four families harbor a common founder mutation. We therefore constructed a haplotype of four SNPs (rs2269672, rs4455261, rs8140949, and rs5761637) and the missense variation (c.493G>C) for a representative affected individual from each of the four families. As shown in Table 2, the affected individuals from each of the four familial cases share a common SNP haplotype that spans >1.4 Mb on chromosome 22q harboring *CRYBB3*, providing suggestive evidence of a common founder effect.

**Table 2:** SNP haplotype of affected individuals of families PKCC025, PKCC063, PKCC131, and PKCC168 harboring the p.Gly165Arg mutation in *CRYBB3*.

Family ID	Individual ID	SNP haplotype				
		rs2269672 (T/C)	rs4455261 (G/A)	*CRYBB3* (c.493G>C)	rs8140949 (G/A)	rs5761637 (T/C)
		Chr 22: 25,201,364	Chr 22: 25,207,051	Chr 22: 25,207,069	Chr 22: 25,231,637	Chr 22: 26,625,493
PKCC025	15	C	G	C	A	C
PKCC063	10	C	G	C	A	C
PKCC131	10	C	G	C	A	C
PKCC168	15	C	G	C	A	C

To confirm the common founder effect, we constructed a haplotype consisting of 10 informative SNPs for one affected individual from each of the four familial cases (S1 Table). In parallel, we retrieved the genotype information of ethnically matched controls from the 1000 Genomes database to estimate population haplotype frequencies (S1 Table). As shown in S2 Table, all families share a common 10 SNP haplotype (CAGTATGCTT). We calculated probabilities that the four familial cases have a common founder. The haplotype-based analysis strongly suggests (p<1.64E-10) that these families share a common founder.

**Fig 6:**
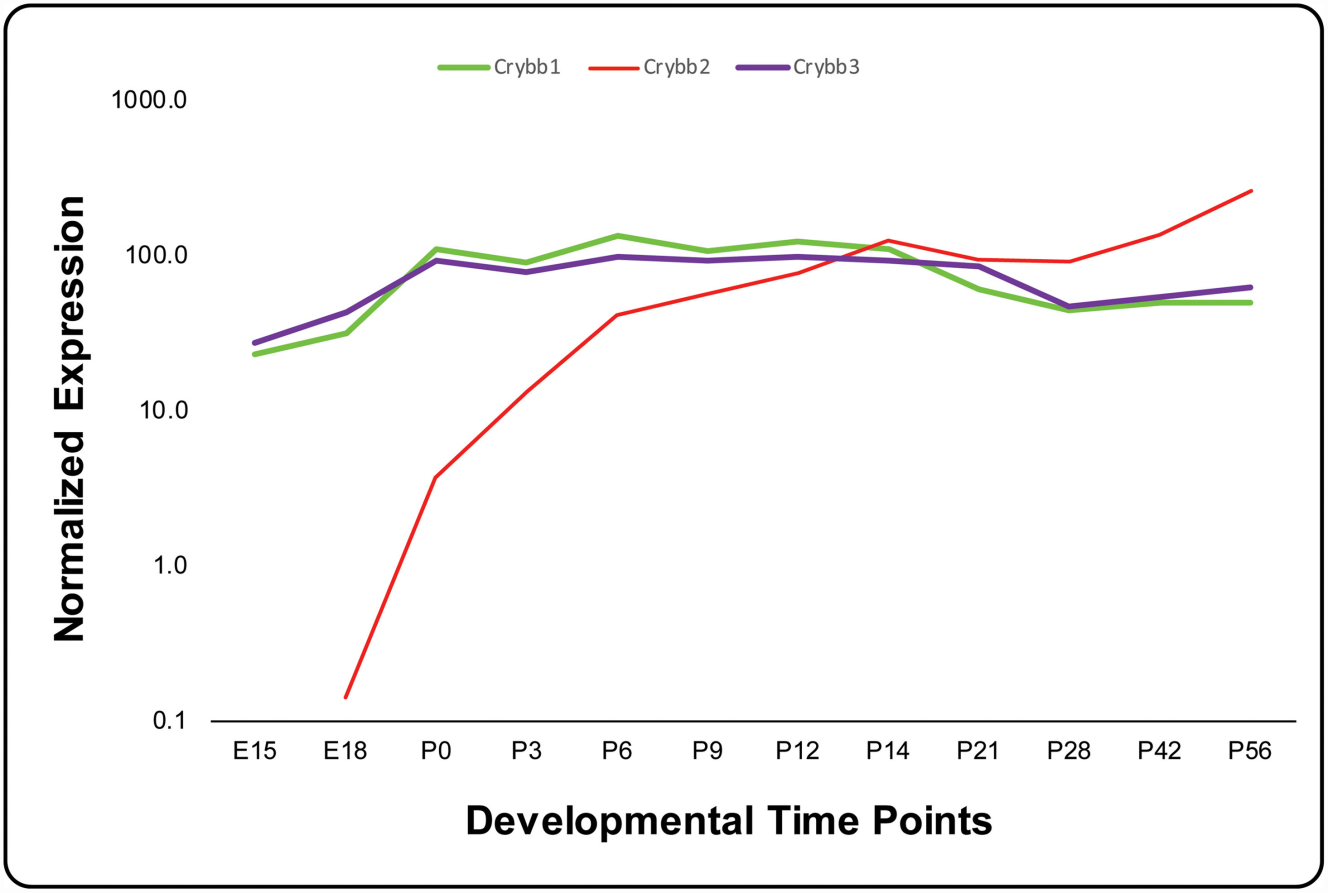
Expression profile of β-crystallin in the developing mouse lens. The expression of *Crybb1*, *Crybb2*, and *Crybb3* at different developmental time points was normalized to *Gapdh*. The x-axis and y-axis represent developmental time points and normalized expression of each mRNA, respectively.

Hawse and colleagues reported *CRYBB3* transcripts are expressed in the lens epithelia, [32] and we recently demonstrated expression of *Crybb3* at six developmental time points in the mouse lens as early as embryonic day 15 (E15) [33]. Our data show a steady expression pattern of *Crybb3* at all six developmental time points including two embryonic and four postnatal stages. We wondered if the expression levels remain consistently high as in the earlier postnatal days or whether they decrease after the initial burst. We examined expression of *Crybb3* in the mouse lens for six additional time points spanning a period of two months using TaqMan assays and compared it with the expression levels of *Crybb1* and *Crybb2*. As shown in Fig 6, the expression levels of *Crybb3* remained relatively steady over 12 developmental time points, similarly to *Crybb1*, and in sharp contrast to *Crybb2* (Fig 6).

## Discussion

Here, we report a common ancestral mutation in *CRYBB3* associated with autosomal recessive congenital cataracts identified in four consanguineous Pakistani families. Initially, a genome-wide linkage scan localized the critical interval to chromosome 22q, followed by localization of three additional families through exclusion analysis. Sanger sequencing identified a causal mutation in *CRYBB3* that was previously reported in two familial cases of same ethnic origin. The mutation segregated with cataracts in all four families. Haplotype analysis strongly suggests that all four families inherited the causal mutation from a common ancestor.

Hulsebos and colleagues mapped *CRYBB3* to chromosome 22q11.2-q12, while Lampi and colleagues confirmed the predicted amino acid sequences by mass spectrometric analysis [28,34]. We previously reported two familial cases of Pakistani origin with nuclear cataracts harboring the G165R missense mutation in *CRYBB3* [21]. Taken together, we have now identified six familial cases harboring the G165R allele of *CRYBB3*, and more interestingly, sharing a common founder.

Since the first report by Riazuddin and colleagues, two additional mutations in *CRYBB3* have been associated with cataractogenesis. Hansen and colleagues reported a missense change in *CRYBB3* (c.224G>A, p.Arg75His) in a Caucasian Italian family, segregating as an autosomal dominant trait with incomplete penetrance [35]. The mutation resides within the second Greek key motif of CRYBB3, substituting a highly conserved amino acid and is therefore most likely pathogenic [35]. Reis and colleagues reported a novel heterozygous missense mutation (c.581T>A, p.Val194Glu) in a patient with bilateral congenital cataracts demonstrating dominant inheritance with reduced penetrance [36]. The mutation resides within the fourth Greek key motif of CRYBB3 and is predicted to be probably damaging by both the PolyPhen2 and SIFT algorithms [36]. Taken together, three causal alleles in *CRYBB3* have been associated with cataractogenesis. Among these, both 75His and 194Glu have been identified only in single familial cases. In sharp contrast, the c.493G>C (p.Gly165Arg) has been identified in six large families and, to date, remains the only variation liable for autosomal recessive cataracts.

Hoang and colleagues recently reported a comparison of lens epithelial cell and lens fiber cell transcriptomes. They reported that *Crybb3* was among the most highly expressed genes in both lens epithelial and fiber cells. The clinical significance of a Gly165Arg substitution would be expected to be significant, given the consistently high levels of CRYBB3 in the ocular lens. Hawse and colleagues reported that many of the genes encoding lens structural proteins exhibited decreased expression in the cataractous lens [32]. This included many of the β- and γ-crystallins, including *CRYBB3*, which are decreased approximately 22-fold [32]. As noted by Hawse and colleagues, it remains to be determined whether the decrease in crystallins is the cause of cataract formation or a secondary effect of cataractogenesis [32].

The transparency of the ocular lens is a distinctive characteristic critical for proper light refraction; nonetheless, the exact mechanism of maintenance of lens transparency is not yet completely understood. Identifying the proteins that are essential for the maintenance of lens transparency is vital to preventing cataractogenesis. The identification of new genes associated with cataractogenesis will help us to understand better the biology of the ocular lens at a molecular level.

## Supporting Information

S1 File ARRIVE Checklist. The ARRIVE checklist has been completed for the manuscript.
(PDF)

S1 Table The SNP genotypes of an affected individual from each of the four families, along with 96 ethnically matched controls from the 1000 Genomes database. The 10 SNPs are rs9608378, rs35420369, rs2283809, rs2252878, rs2252880, rs73162227, rs5760907, rs6519571, rs8142865, rs6004483.
(XLSX)

S2 Table A 10-SNP haplotype of affected individuals of PKCC025, PKCC063, PKCC131, and PKCC168, and 96 ethnically matched controls from the 1000 Genomes database. Haplotype frequencies were estimated using EM and CHM algorithms as implemented in the Golden Helix SVS. **Note:** The 10 SNPs used to construct the haplotype are listed in S1 Table.
(XLSX)
